# Older Adults’ Choice of Patterns of Outdoor Physical Activity Duration: A Mixed Multinomial Logit Model

**DOI:** 10.3390/ijerph18158199

**Published:** 2021-08-03

**Authors:** Zhengying Liu, Wenli Huang, Yuan Lu, You Peng

**Affiliations:** 1 School of Urban Planning & Design, Peking University Shenzhen Graduate School, Shenzhen 518055, China; z.liu@pku.edu.cn; 2 Systemic Change, Department of Industrial Design, Eindhoven University of Technology, P.O. Box 513, 5600MB Eindhoven, The Netherlands; y.lu@tue.nl; 3 Department of Product Design, College of Art and Design, Hubei Engineering University, 272 Jiaotong Road, Xiaogan 432000, China; 4 Urban Planning and Transportation, Department of the Built Environment, Eindhoven University of Technology, P.O. Box 513, 5600MB Eindhoven, The Netherlands

**Keywords:** older adults, physical activity, duration patterns, mixed logit

## Abstract

Outdoor physical activity duration is a key component of outdoor physical activity behavior of older adults, and therefore, an important determinant of their total physical activity levels. In order to develop a successful outdoor physical activity program, it is important to identify any heterogeneity in preferences for outdoor physical activity duration patterns among older adults. In addition, more insight is needed in the influence of environmental characteristics on duration choice for creating supportive neighborhood environments matching individuals’ preferences. To this end, a mixed multinomial logit model is estimated based on one-week data collected among 336 respondents aged 60 or over in 2017 in Dalian, China. The present model formulation accounts for heterogeneity in individuals’ preferences and allows for the analysis of substitution and complementary relationships between the different patterns of outdoor physical activity duration. Results indicate that older adults vary significantly in their preferences for each outdoor physical activity duration pattern. Moreover, short walking duration, short exercise duration and medium exercise duration are substitutes for medium walking duration while short walking duration and short exercise duration are complements for medium exercise duration in terms of individuals’ outdoor physical activity duration preferences. In addition, we find that distance to the nearest park, footpath conditions and neighborhood aesthetics are associated with older adults’ outdoor physical activity duration choice.

## 1. Introduction

Worldwide, the number of older adults aged 60 years or over is expected to grow exponentially from 901 million in 2015 to 2.1 billion by 2050 [1]. This will cause substantial disease burden and pose a major economic burden for societies globally, given age-related diseases and the corresponding healthcare expenditure [2,3,4]. Evidence suggests that regular participation in physical activity provides extensive health benefits in older adults, including decreased risk of coronary heart disease and stroke, diabetes, hypertension, depression and cognitive decline, and increased independent living and improved quality of life [5,6,7]. Older adults, however, are often less physically active. Their prevalence of physical inactivity ranges from about 30% in Southeast Asia to 60% in the Americans [8]. Thus, it is important to identify modifiable factors that may promote more physical activity among older adults. 

As neighborhood characteristics are modifiable and most older adults tend to spend their time engaging in a variety of outdoor activities at some distance from their immediate neighborhoods, understanding the impact of neighborhood characteristics on older adults’ outdoor physical activity is particularly pertinent and many existing physical activity studies have examined older adults’ total outdoor physical activity levels in relation to neighborhood characteristics [9,10,11,12,13]. Specifically, King [14] found that older women who perceived that parks were located within a 20-min-walking distance from their homes showed significantly higher levels of pedometer walking records than women who did not have such facilities. Other researchers found that older adults who perceive the presence of sidewalks/footpaths and enjoyable scenery in the neighborhood are more likely to achieve high levels of outdoor physical activity [15,16,17,18]. Barnett et al. [19] systematically reviewed research into the relationships between environmental attributes and older adults’ total outdoor physical activity levels. They found overall access to destinations and services, aesthetically pleasing scenery and walking-friendly infrastructure are positive environmental correlates of outdoor physical activity levels of older adults. Most previous studies focused on older adults’ differences in outdoor physical activity levels and its relationship to neighborhood characteristics, however, very little research has examined whether older adults differ in specific outdoor physical activity features (e.g., duration, frequency, type, etc.) and their relationships to neighborhood characteristics. In line with “patient-centered care” frameworks [20], a physical activity prescription that incorporates individual preferences, needs or interests in terms of specific aspects of physical activity such as duration, frequency and type may enhance adherence to physical activity [21]. Therefore, an examination of the differences in specific physical activity features is useful to health care providers, as a way to understand how certain features of physical activity prescriptions can be better tailored to meet the physical activity preferences, needs or interests within diverse aging population.

This study specifically focused on older adults’ physical activity duration for at least two reasons. First, physical activity duration was measured at the activity episode level allowing to examine both the intra-individual (e.g., the difference within the same person at different times of the day) and inter-individual variation in physical activity duration. However, other physical activity features such as frequency were usually aggregated at the individual level, only allowing to examine the inter-personal difference. Thus, research on physical activity duration could yield more detailed findings than research on other physical activity features. Second, among a small amount of existing studies on the individual dimensions of physical activity, there are at least several studies on physical activity frequency [22] and patterning of types of physical activity [23]. However, far less attention has been placed on variation in physical activity duration among older adults.

Moreover, a better understanding of the relationship between older adults’ physical activity duration patterns and neighborhood characteristics has practical implications for urban planners and designers in creating neighborhoods tailored to older adults with certain preferred physical activity duration patterns. As individuals with different physical activity duration patterns might have different demands for environmental supportiveness, research needs to identify more accurately the influence of environmental characteristics on the duration choice. This identification is important to inform urban planning and design interventions. In addition, some physical activity duration patterns may be seen as substitutes or complements for each other. On the one hand, research suggests people tend to repeat habitual behaviors [24]. Therefore, those who prefer to conduct walking for 30 min daily might be less likely to conduct walking for 60 min or other types of physical activity for 30 min on a trip. On the other hand, as most older adults have more free time after retirement and they like to spend their free time outdoors as much as possible [25], they are likely to perform multi leisure physical activities during a single tour to fill their increased discretionary time. For example, they are likely to conduct walking for a medium duration and other types of leisure physical activity for a short duration to make their outdoor trips interesting while avoiding the fatigue. Based on the recommended weekly amounts of physical activity [26], older adults can do a combination of different physical activities. However, it is unclear what combination of different physical activities are preferred for older adults. Understanding the substitution and complementarity between different physical activity duration patterns will help health professionals refine physical activity prescription for older adults to yield optimal gains.

Given these aforementioned limitations, the objectives of this study are threefold. First, it is to identify whether there is heterogeneity in preferences for physical activity duration patterns among older adults. We hypothesize that there will be heterogeneity in physical activity duration patterns due to the heterogeneity of socio-demographic characteristics of older adults. Second, it is to investigate the substitution and complementary relationships across physical activity duration patterns. We hypothesize that a medium-duration (i.e., 30–60 min) participation in exercise such as line dancing may complement a short-duration (i.e., 10–30 min) participation in stretching exercise, since some older adults may have higher awareness of the benefits of stretching warm-up before exercise or want to fill their increased discretionary time through participation in a variety of leisure activities. We also expect that a medium-duration participation in one activity may substitute a short-duration participation in the same activity, because older adults tend to repeat their habitual/preferred behavior routinely. Third, it is to examine the influence of neighborhood characteristics on the duration choice controlling for effects of individuals’ socio-demographic characteristics and specific aspects of the activity episodes such as timing of participation. We hypothesize that significant associations of physical activity duration patterns and neighborhood characteristics will be observed among older adults since they face in general a declining health and increasing mobility limitations [27] and thus likely are more dependent on the neighborhood environment.

This study makes three contributions to the literature. Firstly, we expand the research on older adults’ physical activity by examining the differences in physical activity duration patterns rather than just a summary score of physical activity levels which ignores the differences in specific physical activity features among older adults. Secondly, we explore the yet under-investigated substitution and complementary relationship between physical activity duration patterns. Thirdly, to the best of our knowledge, the current study is the first study that examines the relationship between neighborhood characteristics and physical activity duration patterns, using detailed disaggregated measures of physical activity. 

## 2. Materials and Methods

### 2.1. Data Source and Collection

The data used for this study were collected between August and September 2017 in diverse neighborhoods in Dalian, China. A pilot survey preceded the main survey in order to detect and correct different problems with the questionnaire and its administration. Five assistants from the Dalian University of Technology were recruited and trained about basic communication skills, procedures and the used questionnaire to administer the survey. The neighborhoods were purposively selected from three location categories, namely, the inner city, the fringe of the city and the area between the inner city and the fringe, in order to have variations with respect to the neighborhood environmental characteristics. Then in each area, residents aged 60 or over were approached personally. Considering the fact that different older people might have different preferences for outdoor activity locations and inclusion of such diverse older people is important to reduce sampling bias, participants were recruited from different outdoor locations such as streets, local squares and parks, etc. Those who are eligible and agreed to participate were asked to answer a series of questions about their weekly routine outdoor activity behaviors, socio-demographic characteristics, and perceptions of neighborhood environments. All subjects gave their informed consent for inclusion before they participated in the study. The study was conducted in accordance with the Declaration of Helsinki. The questions were asked in Chinese but were translated for the purpose of this manuscript.

### 2.2. Measures and Instruments

Regarding routine outdoor activity behaviors, an interviewer-administered questionnaire involving a 7-day recall was used. In the questionnaire, detailed information was gathered about each activity episode. This information includes the start time, origin, destination, trip duration, duration of the activity episode, etc. For further details about the data collection procedure we refer to Liu et al. [28].

The process of developing the sample for this study involved several steps. First, individuals who participate in outdoor leisure-time physical activity in a typical week were selected from the larger group of the population, that is, a valid sample of 363 respondents. Second, their outdoor weekly leisure-time physical activities and travel episodes were selected from the larger activity survey database. Finally, the socio-demographic and environmental characteristics were appended to each activity episode based on the ID number of the respondents. The final sample for analysis includes 4487 weekly outdoor leisure-time physical activity episodes of 336 individuals.

Data on gender, age, education level, and income level were obtained using a socio-demographic questionnaire. A follow-up questionnaire was used to ask participants to evaluate their local area by responding to statements concerning various environmental characteristics. The subscales employed in this study include the following: footpath conditions and neighborhood aesthetics. Drawing on the Environmental Module of the International Physical Activity Questionnaire (IPAQ-E) [29] and the questionnaire for residential satisfaction [30], two single-item measures were used and respondents were asked to indicate their extent of satisfaction with footpath conditions and neighborhood aesthetics on a five-point Likert scale. In addition, distance to the nearest park was objectively measured using ArcGIS combined with Baidu Map, and the distance measure was taken using network distances.

### 2.3. Model Specification

The dependent variable in the analysis is the choice of the pattern of outdoor physical activity episode duration. As the dependent variable is a categorical variable, generally, a multinomial logit model [31] could be used. However, it assumes that the parameters of the utility function are the same for everyone, which seems unrealistic in this case. Moreover, it does not consider any unobserved factors that persist over time for a given decision maker with repeated choices. Given this fact, we chose a mixed multinomial logit model [32,33,34,35] which have three advantages relative to the standard multinomial logit model. First, the mixed logit provides a better representation of the choice situation than a standard logit, as it accommodates unobserved heterogeneity across individuals by allowing the parameters associated with each variable to vary randomly around a mean. In the present study, unobserved heterogeneity implies that different individuals have different preferences for physical activity duration patterns. Second, the mixed logit model provides more information (i.e., the relationships between different duration pattern choices) than a standard logit, as it considers correlation in common unobserved factors influencing the choice of duration patterns. For example, unobserved attributes such as a personality characterized by lazy may increase the utility of short walking duration while decrease the utility of medium walking duration. Third, the mixed logit model aligns itself more with reality than a standard logit, which is beneficial to provide better estimation accuracy, as it accommodates individual-specific unobserved factors that influence repeated choices of the same individual (one trip during the week was considered a choice situation in our study). In addition, although the mixed multinomial logit demands better quality data than the multinomial logit model, our dataset consists of 4487 physical activity episodes capturing a great amount of true behavioral variability and therefore supports the application of the mixed multinomial logit model.

Specifically, we formulate a mixed multinomial logit model of physical activity duration for the choice among five patterns: (1) short exercise duration (1–30 min), (2) medium exercise duration (31–60 min), (3) short walking duration (1–30 min), (4) medium walking duration (31–60 min), and (5) long walking duration (60+ min). The duration of physical activity was classified into three categories namely 1–30 min, 31–60 min and 60+ min for two reasons. First, our preliminary analysis showed that the duration of physical activity of older adults was distributed discretely with several peaks concentrated around certain cutoff values which are integral multiples of 10 min such as 10, 30, 60 min. Second, the Physical Activity Guidelines Advisory Committee divided the amount of physical activity an adult gets every week into four categories: inactive (no activity), low (fewer than 150 min), medium (150 min to 300 min) and high (more than 300 min) [7] and the recommended frequency for moderate-intensity physical activity is usually five days per week [36]. Therefore, we selected three cutoff values namely 30 min and 60 min and considered 1–30 min of physical activity as short duration, 31–60 min as medium duration and 60+ min as long duration.

The model used in this study can be described as follows. An individual *i* chooses among *J* possible alternatives. The utility that individual *i* derives from alternative *j* at observation *t* is:

(1) $$U_{ijt}=\alpha_{ij}^{*}+\beta_{jk}X_{ikt}+\sigma_{jg}G_{igt}+\theta_{jh}H_{iht}+\omega_{ju}U_{iut}+\gamma_{jw}W_{iwt}+\delta_{js}S_{ist}+\mu_{jp}P_{ipt}+\varepsilon_{ijt\;}$$

The first term $\alpha_{ij}^{*}\;$ is a random alternative-specific constant associated with individual i and activity duration alternative *j* (*j* = 1, 2, 3, 4). For the reference level of long walking duration (*j* = 5), the utility is equal to zero. The value of each random parameter $\alpha_{ij}^{*}$ is drawn from a normal distribution with a particular mean and standard deviation. A normal distribution is assumed as we expect preferences to deviate from the mean in both directions equally, that most deviations will be around the mean, and that extreme deviations occur less frequently. This means that for the constants (patterns of outdoor activity duration) not only mean coefficients were estimated, but also the standard deviations. This approach allows the estimation of the distributions for each of these outdoor physical activity duration patterns (except for the reference level), providing a rich array of preference information. In addition, the selection of the normal distribution of random parameters was confirmed by comparison with the parameter estimates following other distributions such as the lognormal, Weibull distribution, etc.

The other components represent vectors of non-random parameters of episode participation occasion variable, individual socio-demographic and neighborhood environmental characteristics. The coefficients that will be estimated respectively are duration of indoor activity (*β*), gender (*σ*), age (*θ*), income level (*ω*), distance to the nearest park (*γ*), footpath condition (*δ*), and neighborhood aesthetics (*μ*). The subscripts *k, g, h, u, w, s*, and *p* represent the levels of each of the variables just cited respectively. Finally, $\varepsilon_{ijt\;}$ is an unobserved random term assumed to be independent from the other components.

## 3. Results

Table 1 shows the characteristics of the sample and environmental attributes of neighborhoods. In order to reduce the model complexity, most of the independent variables were re-coded from five to two categories. As can be seen, the sample is almost equally divided by gender. They are also almost equally distributed over the age categories from 60 to 80+ years of age. 60.5% of the respondents have medium education or lower. With respect to individual income level, 52.6% of the sample indicates that they have an income of less than CNY3000 per month. Regarding neighborhood environmental characteristics, the results show that 74.1% of the sample lives in an area with a distance of over 800 m to the nearest park. The majority of the respondents (85.4%) are (very) satisfied with footpath conditions and 47.0% are (very) satisfied with neighborhood aesthetics.

The dependent variable in the analysis is the choice of the pattern of outdoor physical activity episode duration. This choice is characterized by five alternatives: short walking duration, medium walking duration, long walking duration, short exercise duration and medium exercise duration. The independent variables aiming to explain activity duration choice include episode participation occasion variable, socio-demographic characteristics and neighborhood environmental characteristics. They were entered in the model as non-random parameters. All the categorical explanatory variables are effect coded. This means that for a variable with L categories, L-1 indicator variables are created. Each category corresponds with a value of one on the corresponding indicator variables and a value of zero on all other indicator variables. The reference category is coded as -1 on all indicator variables. Consequently, all estimated coefficients sum to zero and estimated parameters can be interpreted as deviations from the mean.

The mixed multinomial logit model was estimated using the econometric software, NLOGIT version 5.0 (Econometric Software, Inc., Plainview, NY, USA). Five hundred Halton draws were used to estimate the parameters of the model. The estimated mixed multinomial logit model resulted in a McFadden pseudo Rho-square of 0.579 which indicates a good model fit.

The final specification results of the outdoor physical activity episode duration pattern choice model are presented in Table 2. In the following sections, we discuss the effect of variables by variable category. The observed variables are included with the coefficient on long walking duration pattern considered as the base (i.e., the coefficient on long walking duration is arbitrarily normalized to zero).

The standard deviations of the parameter distributions of the physical activity duration patterns are all significant at the 1% statistical level, indicating that there is significant intra and inter individual variation due to unobserved factors affecting physical activity duration pattern choice. Parameters indicate higher preferences for medium walking duration, followed by short walking duration, medium exercise duration and short exercise duration. The estimated unobserved covariance between physical activity duration pattern alternatives is all significant. It suggests that the unobserved factors that increase the probability of participation in medium duration walking decrease the propensity to participate in short duration exercise and medium duration exercise. In other words, medium walking duration is a substitute for short exercise duration and medium exercise duration. On the contrary, factors that increase the probability of participation in medium duration exercise (i.e., line dancing) also increase the propensity to engage in short duration exercise (i.e., stretching). These activity duration patterns complement each other. In addition, medium walking duration is a substitute for short walking duration. This might imply that those who have higher preferences for medium duration walking are less interested in short duration walking and are more likely to repeat their habitual/preferred behavior routinely. The duration of indoor activity is positively associated with short walking duration, short exercise duration and medium walking duration, compared to long walking duration.

Several individual characteristics were tested in the model, but only those related to gender, age and income level appeared in the final specification. Education is not included in the model as it is strongly correlated with income (*r* = 0.604, *p* < 0.001). The results indicate that older males are more likely to conduct medium duration walking compared to older females. The effect of age indicates that individuals in the 80+ group are more likely to participate in short duration walking, exercise and medium duration walking while less likely to participate in medium duration exercise. Individuals in the 75–79 age groups have higher preferences for engagement in medium duration exercise. Preferences for activity duration differ between income levels. Estimated coefficients indicate that individuals in the group of high-income level have lower preferences for short to medium duration walking compared to long duration walking. 

Among the neighborhood environmental characteristics, the effect of distance to the nearest park indicates that shorter distance to the nearest park is negatively associated with the probability of participation in short and medium duration walking, compared to long duration walking. Results for footpath conditions show that individuals who are satisfied with footpath conditions are more likely to participate in any type of outdoor physical activity. The coefficients of neighborhood aesthetics indicate that individuals who are satisfied with neighborhood aesthetics are more likely to participate in short duration walking, exercise and medium duration exercise. 

## 4. Discussion

The study presents a model for the pattern of outdoor physical activity episode duration that individuals pursue in a typical week. The choice set characterizing the pattern of outdoor physical activity duration includes short exercise duration, medium exercise duration, short walking duration, medium walking duration and long walking duration. The focus on the pattern of outdoor physical activity duration is motivated by (a) an interest in investigating heterogeneity across individuals in the preferences for various outdoor physical activity duration patterns, (b) an interest in examining substitution and complementary relationships between outdoor physical activity duration patterns, and (c) a desire to examine the determinants of choosing specific outdoor physical activity duration patterns that contribute to develop effective environmental interventions tailored to older adults’ preferred or habitual outdoor physical activity duration patterns and then promote healthy aging.

The results of the model suggest that older adults prefer medium walking duration the most followed by short walking duration, medium exercise duration and short exercise duration. The results also show significant heterogeneity in the respondents’ preferences for various physical activity duration patterns. In terms of substitution and complementary relationships between physical activity duration patterns, it was found that medium duration exercise increases the propensity to perform short duration walking and exercise. Medium duration walking decreases the probability of participation in short and medium duration exercise. Unobserved factors that increase the likelihood of participation in medium duration walking decrease the propensity for participation in medium duration exercise. The duration of indoor activity is positively associated with short walking duration, short exercise duration and medium walking duration, compared to long walking duration. Overall, these findings support the argument that with limited time to participate in activities, there may be substitution between activity episodes [37,38]. The updated physical activity guidelines [26] recommended that older adults can do a combination of moderate- and vigorous-intensity physical activities to achieve health benefits. However, our study found that medium duration walking which tends to be moderate intensity is a substitute for medium duration exercise which mainly refers to line dancing in this study and tends to be vigorous intensity. This implied that doing a combination of moderate- and vigorous-intensity physical activities might not be preferable for older adults, although they could achieve health benefits by doing it. Therefore, encouraging older adults to do a combination of moderate- and vigorous-intensity physical activities is not recommended at least from the perspective of behavior habits.

With respect to socio-demographics, we found that older men are more likely to engage in medium duration walking, while older women are more likely to participate in medium duration exercise (i.e., line dancing). Given that line dancing is popular among Chinese older women [39], the findings are plausible. However, in contrast to these results, Branion-Calles et al. [40] found that walking was more common among women. Individuals aged 80 years or over have a higher likelihood of engagement in short to medium duration walking compared to long duration walking. These findings are plausible as older people’s fatigue endurance declines with age [41] and older elders are more likely to feel tired than younger elders during long-time walking. Individuals with high income level are less likely to perform short or medium duration exercise compared to long duration walking. This finding is in line with Liu et al. [22] who found that high-income individuals tend to be physically active.

Regarding neighborhood environmental characteristics, our study found that older adults with easier access to parks are less likely to participate in short and medium duration walking, compared to long duration walking. This is, to some extent, in line with previous findings indicating that access to parks are positively associated with levels of total walking of older adults [10,42,43]. This might imply that one mechanism by which access to parks affects total walking is by influencing individuals’ duration of participation in walking. Older adults who are satisfied with footpath conditions are likely to participate in any type of outdoor physical activity. In other words, the level of satisfaction with footpath condition does not lead to the difference in physical activity duration. This might partly explain the finding that there is no association between footpath quality and levels of total physical activity and total walking [19]. Individuals who are satisfied with neighborhood aesthetics have a higher propensity to participate in short and medium duration exercise. Nevertheless, neighborhood aesthetics has no relationship with medium walking duration, which is inconsistent with earlier studies demonstrating that neighborhood aesthetics plays an important role in facilitating walking [44,45,46]. Our results might imply that neighborhood aesthetics would be more important for engaging in duration type-specific leisure-time exercise than for walking. Overall, our results provide preliminary evidence for the idea that environmental characteristics affecting the frequency of physical activity might have no influences on the duration of physical activity and those affecting the duration of one physical activity might not influence the duration of other physical activities. In future research it would be of interesting to do a systematic research on whether different environmental correlates exist for the different components (i.e., frequency and duration) of different physical activities.

The above results have important implications for neighborhood design and public health. First, this research demonstrates that older adults vary in their preferences for each physical activity duration pattern, implying that the components (i.e., duration) of a successful physical activity program should be tailored to meet the physical activity duration needs and interests within the diverse population in order to increase adherence to physical activity. In other words, an appropriately designed physical activity program would be more successful than a vague one without considering older adults’ different needs and preferences for activity duration patterns. 

Second, our research suggests that individuals aged 80 or over have a higher preference for participation in short duration walking and exercise, which might be due to the age-related decline in ability to perform long duration activities. This implies that higher levels of physical activity for this age group might be achieved only through the frequent participation in short duration activities. In other words, interventions aimed at frequency of physical activity may be more effective than those aimed at episode duration in enhancing physical activity levels of the population aged 80 or over. Projections indicate that in 2050 the older adults aged 80 years or over will number 434 million, having more than tripled in number since 2015 [1]. This requires more attention and effort devoted to maintaining their health and quality of life. Thus, it would be necessary and interesting, for future research, to see what environmental characteristics have positive effects on the frequency of participation in physical activities of such subgroup of older adults.

Third, this research indicates that older females have a higher preference for medium duration exercise (i.e., line dancing) and demonstrates the importance of neighborhood aesthetics in supporting medium duration exercise. Providing a variety of outdoor fitness equipment in the neighborhood along with making areas more attractive by planting trees and flora, etc. [19], might therefore be a valuable strategy to promote physical activity and health among older men.

There are some limitations of this study. Firstly, the measures of physical activity relied on self-reports which are often subject to recall bias. However, we used a guided memory technique in which interviewees were encouraged to think of their typical week as a continuous series of episodes in a film and then to answer structured questions about each episode in chronologic order. As this technique has been shown to be beneficial to provide a more accurate recall [47], the recall bias is considered small. Secondly, as the study focused only on older adults who conducted outdoor leisure-time physical activity, its findings might not be generalizable to those who did not conduct outdoor physical activity. Thirdly, this study did not control for the effect of activity intensity on duration of activity, which is likely to lead to estimation biases. Future research should take it into account. Finally, this study focused solely on the duration of physical activity. For future research, it would be of interest to do systematic research on the duration, frequency and type of physical activity and their relationships with neighborhood characteristics.

## 5. Conclusions

This study suggested that older adults vary significantly in their preferences for different outdoor physical activity duration patterns. Short walking duration, short exercise duration and medium exercise duration are substitutes for medium walking duration while short walking duration and short exercise duration are complements for medium exercise duration in terms of individuals’ outdoor physical activity duration preferences. Moreover, the results indicated that distance to the nearest park, footpath conditions and neighborhood aesthetics are associated with older adults’ outdoor physical activity duration choice. Having a better understanding of the outdoor physical activity duration pattern among older adults have important implications for developing tailored interventions to promote outdoor physical activity participation of this population. 

## Figures and Tables

**Table 1:** Sample characteristics (*n* = 336).

Variables	Levels	*n*	%
Socio-demographics			
Gender	Male	169	50.3
	Female	167	49.7
			
Age (years)	60–64	72	21.4
	65–69	70	20.8
	70–74	70	20.8
	75–79	55	16.4
	80+	69	20.5
			
Education level	Middle school or lower	203	60.5
	High school to college/university	133	39.5
			
Income level	Low to medium(≤CNY 3000 per month)	177	52.6
	High(>CNY 3000 per month)	159	47.4
			
Built environmental characteristics			
Distance to the nearest park	0–800 m	87	25.9
	800+ meters	249	74.1
			
Footpath conditions	Less satisfied	49	14.6
	(Very) satisfied	287	85.4
			
Neighborhood aesthetics	Less satisfied	178	53.0
	(Very) satisfied	158	47.0

CNY = Chinese Yuan.

**Table 2:** Model results.

Variable	Coefficient (95% CI) (*SE*)			
	Short Walking Duration	Medium Walking Duration	Short Exercise Duration	Medium Exercise Duration
Random parameters				
Constant	−1.514 *** (−2.358, −0.670) (0.431)	0.531 (−0.361, 1.422) (0.455)	−4.670 *** (−5.847, −3.493) (0.601)	−2.552 *** (−3.730, −1.374) (0.601)
Standard deviation	8.984 *** (7.835, 10.132) (0.586)	10.273 *** (8.898, 11.648) (0.701)	6.222 *** (5.407, 7.036) (0.416)	9.832 *** (8.902, 10.762) (0.475)
Unobserved covariance				
Medium walking duration	−12.552 *** (−18.767, −6.337) (3.170)			
Short exercise duration	22.215 *** (13.743, 30.686) (4.322)	−20.911 *** (−25.342, −16.481) (2.260)		
Medium exercise duration	36.488 *** (26.488, 46.489) (5.102)	−32.936 *** (−36.617, −29.256) (1.878)	6.748 *** (2.289, 11.208) (2.275)	
Non-random parameters				
Episode participation occasion variable				
Duration of indoor activity	0.013 *** (0.010, 0.016) (0.002)	0.004 *** (0.001, 0.007) (0.001)	0.023 *** (0.019, 0.026) (0.002)	−0.003 (−0.007, 0.000) (0.002)
Individual socio-demographic characteristics				
Gender				
Male	−0.311 *** (−0.679, 0.057) (0.188)	0.907 *** (0.431, 1.384) (0.243)	0.106 (−0.411, 0.622) (0.263)	−2.168 *** (−2.687, −1.649) (0.265)
Female	0.311	−0.907	−0.106	2.168
Age				
60–64	−2.598	−3.277	−1.636	−2.909
65–69	0.571 (−0.547, 1.690) (0.571)	0.698 (−0.416, 1.812) (0.568)	1.201 * (−0.023, 2.424) (0.624)	0.972 (−0.203, 2.147) (0.599)
70–74	0.635 * (−0.056, 1.325) (0.352)	0.317 (−0.356, 0.991) (0.344)	−1.024 *** (−1.772, −0.276) (0.382)	−0.019 (−0.744, 0.705) (0.370)
75–79	−0.254 (−0.991, 0.484) (0.376)	0.642 * (−0.067, 1.352) (0.362)	0.137 (−0.685, 0.959) (0.419)	2.607 *** (1.767, 3.447) (0.429)
80+	1.646 *** (0.825, 2.467) (0.220)	1.620 *** (0.717, 2.523) (0.460)	1.322 *** (0.350, 2.294) (0.496)	−0.651 (−1.766, 0.464) (0.569)
Income level				
Low to medium	1.612	2.132	−1.270	0.123
High	−1.612 *** (−2.043, −1.181) (0.220)	−2.132 *** (−2.577, −1.687) (0.227)	1.270 *** (0.520, 2.021) (0.232)	−0.123 (−0.515, 0.269) (0.200)
Environmental characteristics				
Distance to the nearest park				
0–800 m	−0.581 *** (−0.959, −0.202) (0.193)	−1.133 *** (−1.548, −0.717) (0.212)	0.506 * (−0.015, 1.028) (0.266)	−0.472 (−0.824, −0.119) (0.180)
800+ meters	0.581	1.133	−0.506	0.472
Footpath condition				
Less satisfied	−2.571	−2.508	−0.739	−1.396
(Very) satisfied	2.571 *** (1.938, 3.204) (0.322)	2.508 *** (−1.549, −0.717) (0.305)	0.739 ** (−0.015, 1.028) (0.303)	1.396 *** (0.853, 1.938) (0.277)
Neighborhood aesthetics				
Less satisfied	−2.140	−0.186	−1.212	−0.572
(Very) satisfied	2.140 *** (1.704, 2.577) (0.223)	0.186 (−0.175, 0.547) (0.184)	1.212 *** (0.783, 1.641) (0.219)	0.572 *** (0.238, 0.906) (0.170)
Goodness of fit statistics				
Log likelihood (null model)			−7221.54791	
Log likelihood (estimated model)			−3039.5805	
McFadden Rho−square			0.579	

CI = confidence intervals; *SE* = standard errors. ***, **, * mean significant at 1%, and 10% level.

## Data Availability

The data presented in this study are not publicly available due to privacy.
